# ARCHITECTURAL, INTERIOR DESIGN, AND TECHNOLOGICAL INTERVENTIONS TO FACILITATE REHABILITATION AND AGING IN PLACE

**DOI:** 10.1093/geroni/igad104.3179

**Published:** 2023-12-21

**Authors:** Swati Katyarmal, Michelle Wyndham-West, Shehroz Khan

**Affiliations:** OCAD University, Toronto, Toronto, Ontario, Canada; OCAD University, Toronto, Toronto, Ontario, Canada; Toronto Rehabilitation Institute, Toronto, Ontario, Canada

## Abstract

Most older adults 60 years and above live in the community with either support from their caregivers or independently. Aging in place successfully involves safely carrying out activities of daily living at home without accidents and injuries. However, most traditional homes are not equipped with safe interior designs or support technological interventions, which could be a barrier to aging in place. We performed a narrative review to understand the importance of incorporating architectural, interior design, and technological interventions to support the rehabilitation of older adults with disabilities to successfully age in place. We reviewed 28 research papers from several research databases matching the inclusion criteria on interventions targeting older adults’ rehabilitation outcomes. We included studies that focused on various health conditions (e.g., spinal cord injury, stroke) and supporting home modification along with technological interventions. The review shows that the home environment has a significant impact on older adults’ overall health, and recovery, including functional capacities, cognitive processes, and emotional states providing autonomy, safety, and accessibility. Several safe design features were found, including barrier-free entrance, mobile stair climber, handrails and grab bars, anti-skid flooring, open layout, and shower seat. The integration of technologies (e.g., sensor lights, voice-activated devices) was identified as a promising avenue for enhancing the quality of life. Co-designing the built space with older adults was found to be effective. Our review highlights the need for collaboration between healthcare professionals, engineers, architects, and policymakers to design existing or new homes that support safe aging in place of older adults.

